# Supplementary material for: Assembling a global health image: Ethical and pragmatic tensions through the lenses of photographers
Source: PLOS Glob Public Health. 2024 Feb 14;4(2):e0002540. doi: 10.1371/journal.pgph.0002540 (PMC10866471; doi:10.1371/journal.pgph.0002540)
Supplement: Attachment — Submitted filename: Letter to peer reviewers v2.pdf [file pgph.0002540.s001.pdf]

Dear Peer Reviewers,

Thank you very much for your feedback and an opportunity to make the article stronger. As such, we nearly doubled the empirical part to showcase more problems and challenges through the lenses of the photographers, and slightly adjusted the title. We removed the empirical section about the local photographers-- we decided it will be another article.

Here is the summary of changes in accordance with your comments:

First, we did our best to adjust the overly-ambitious tone where possible, especially in the introduction and discussion in accordance with the concrete sentences you spotted.

Second, as a primary author I did my best to be as detailed and precise as possible in showcasing the materials and methods, and highlighting the fact that I was the person who conducted interviews and was the only person in the group who had access to the data set. I agree with you that the previous version contained an unsatisfactory description of such a pivotal article component.

Third, we added two sections that didn't exist before: (1) conclusion, and (2) limitations & self-reflexivity.

Fourth, we did our best to define all the terms that could be unfamiliar to readers (tropes, poverty porn, etc) and added our justifications for using the common terms such as Global North and South. Please find them in the materials and methods section.

Also, I made it more explicit that I re-contacted the informants about the use of their sensitive quotes presented in this article.

Please let us know if there is anything else we could do to improve this article.

Thank you very much!

-The Lead author and on behalf of the team.
